# Understanding of Plant Salt Tolerance Mechanisms and Application to Molecular Breeding

**DOI:** 10.3390/ijms252010940

**Published:** 2024-10-11

**Authors:** Yuxia Zhou, Chen Feng, Yuning Wang, Chunxia Yun, Xinqing Zou, Nuo Cheng, Wenping Zhang, Yan Jing, Haiyan Li

**Affiliations:** School of Breeding and Multiplication (Sanya Institute of Breeding and Multiplication), Hainan University, Sanya 572025, China; 20223006951@hainanu.edu.cn (Y.Z.); chenfeng@hainanu.edu.cn (C.F.); 20223006829@hainanu.edu.cn (Y.W.); 20213007038@hainanu.edu.cn (C.Y.); 20223006694@hainanu.edu.cn (X.Z.); 20223007083@hainanu.edu.cn (N.C.); 996107@hainanu.edu.cn (W.Z.)

**Keywords:** salinity, salt response, salt signaling regulation, plant salt tolerance, molecular breeding

## Abstract

Soil salinization is a widespread hindrance that endangers agricultural production and ecological security. High salt concentrations in saline soils are primarily caused by osmotic stress, ionic toxicity and oxidative stress, which have a negative impact on plant growth and development. In order to withstand salt stress, plants have developed a series of complicated physiological and molecular mechanisms, encompassing adaptive changes in the structure and function of various plant organs, as well as the intricate signal transduction networks enabling plants to survive in high-salinity environments. This review summarizes the recent advances in salt perception under different tissues, physiological responses and signaling regulations of plant tolerance to salt stress. We also examine the current knowledge of strategies for breeding salt-tolerant plants, including the applications of omics technologies and transgenic approaches, aiming to provide the basis for the cultivation of salt-tolerant crops through molecular breeding. Finally, future research on the application of wild germplasm resources and muti-omics technologies to discover new tolerant genes as well as investigation of crosstalk among plant hormone signaling pathways to uncover plant salt tolerance mechanisms are also discussed in this review.

## 1. Introduction

**Figure 1:**
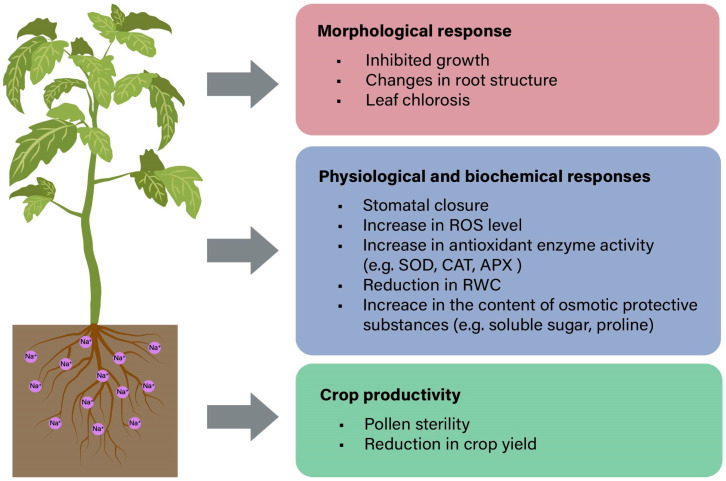
A series of adverse effects in plants induced by salt stress.

Soil salinity is one of the major obstacles to soil productivity, which adversely affects plant growth and development, and ultimately reduces crop yields [1] (Figure 1). Approximately 20–50% of the irrigated land worldwide is affected by salt stains [2]. Due to the global climate change and human activities, the area of saline soil continues to increase by 10% per year. It is estimated that by 2050, more than half of the world’s arable land is predicted to be salinized [3]. Soil salinization may be caused by water shortage and sea level rise, as well as by improper irrigation with high salinity groundwater for a long time [4,5]. Generally, the salinized soils possess considerable amounts of salts including Na^+^, Cl^−^, SO_4_^2−^ and HCO_3_^−^, and some other inorganic substances can also accumulate to larger quantities. When the electrical conductivity (EC) of the soil sample in the selected area exceeds 4 dS/m, and the exchangeable Na^+^ content is above 15%, it can be defined as saline soil [6]. High concentrations of Na^+^ and Cl^−^ in saline soil can prevent plants from absorbing water and nutrients [7]. In addition, saline soil is generally poorly ventilated, so the plants may struggle with hypoxia, which retards their own growth [8]. 

Plants show differences in their capacity to withstand the elevated salinity and are commonly categorized as four classes: salt sensitive, moderately salt sensitive, moderately salt tolerant and salt tolerant [9]. The salt-tolerant plants with the ability of surviving in high salt concentrations (>200 mM NaCl) are known as halophytes. These species have unique structures, such as epidermal bladder cells that store an excessive amount of Na^+^ in their vacuoles, which allows them to adapt to high salinities [10]. Halophytes are considered as useful resources for identifying gene loci and natural variants crucial for plant resistance to salt stress [11]. However, most staple crops (such as rice, wheat, maize and soybean) are glycophytes, which are salt sensitive and incapable of adjusting to soils containing concentrations of NaCl above 200 mM [12]. Nowadays, with the rising world population, people’s demand for food is also increasing, which urges us to optimize the salinity stress tolerance of crops in order to improve crop yields on salinized farmland. Therefore, it is imperative to comprehend the mechanisms of how plants respond to and adapt to salt stress, and in recent decades, a great deal of work has been achieved in this field.

Salt stress can produce many negative effects on plants, such as growth and development inhibition, ionic imbalance, osmotic stress and secondary damage [11]. Plants have developed well-established mechanisms to respond to salt stress, which encompass sensing, complex signaling and the initiation of salt tolerance mechanism [13,14]. Specifically, to adapt to stressful environments, plants need to adjust their physiological and biochemical processes (Figure 1). They sense and respond to salt stress through complex signaling regulatory networks, including calcium signaling, hormone signaling, reactive oxygen species (ROS) signaling and phosphatidic acid (PA) signaling, thereby activating a series of tolerance mechanisms [15,16]. Understanding these mechanisms provides the necessary information for improving crop salt tolerance through molecular breeding. In this review, we primarily discuss the tissue specificity adjusting with salt tolerance, the physiological response of plants to salt stress and the research advancements of the signal regulatory network induced by salt stress. We also summarize the basic strategies for the development of salt-tolerant crops via multi-omics and genetic engineering approaches and present the possible strategies and applications of important determinants in breed improvements and molecular breeding for enhancing plant salt tolerance in the future. 

## 2. Salt Responses under Different Tissues and Organs of Plants

### 2.1. Perception and Response of Salt Stress by Roots

The root is the first organ to perceive salt stress in the soil, which is divided into the primary root (PR) and lateral root (LR) based on where it originates [17,18]. The surface of PRs and LRs is covered by numerous root hairs that are responsible for absorbing nutrients and water. Salt alters the architecture of the root by influencing the quantity and elongation of PRs, LRs and root hairs, causing serious negative effects on root development [19].

**Figure 2:**
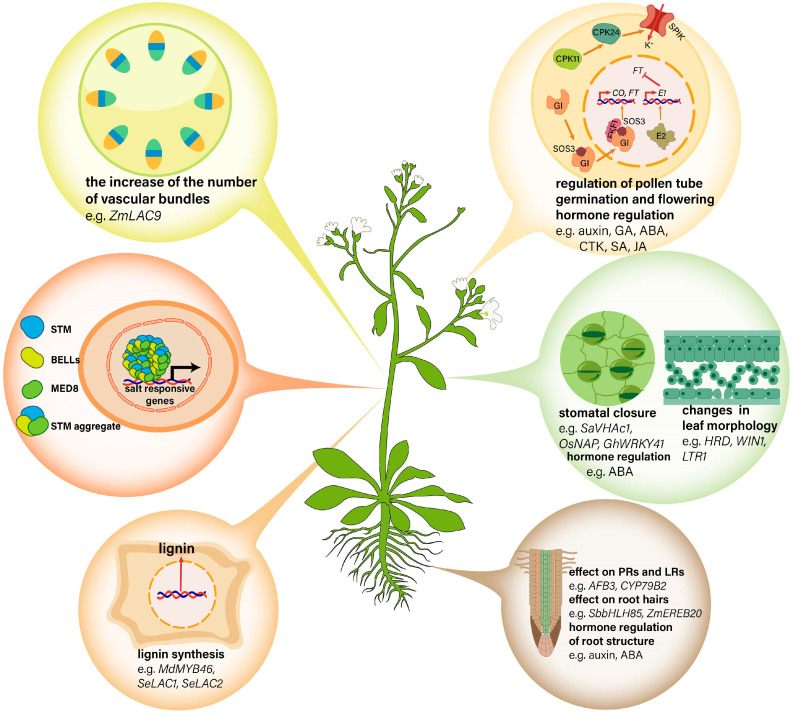
Salt responses in different tissues and organs of the plant.

To date, many genes have been identified as regulating the development of PRs, LRs and root hairs in response to salt stress (Figure 2). Overexpression of *AFB3* (auxin signaling F-Box 3 receptor) of *Arabidopsis* enhances plants’ tolerance to salinity with regards to root architecture and germination [20]. Under salt stress, the formation of LRs is associated with cytochrome P450 family 79 subfamily B2 (CYP79B2), which is also linked to the average length of LRs relative to PRs. Furthermore, in *cyp79b2/cyp79b3* double mutants, the number and length of LRs significantly decreases [21]. Sorghum *SbbHLH85*, a basic helix-loop-helix (bHLH) member, improves salt tolerance by promoting root hair development [22]. Additionally, overexpressing the maize AP2-ERF family member, *ZmEREB20*, can rescue the inhibition of salt stress on *Arabidopsis* root hair growth [23]. It is noted that plant hormones also play an important role in regulating root architecture upon salinity. The phenomenon of halotropism, caused by the root escape from salt stress through bending reflection, is mainly achieved by the asymmetric distribution of auxin efflux carriers at the root tip [24]. On the side of the root with the higher salt concentration, PIN-FORMED2 (PIN2) internalizes in response to salt gradient exposure, which causes auxin redistribution and root structure reshaping [25]. Moreover, salt stress controls the growth of rice PRs through abscisic acid (ABA) accumulation. This is mainly because ABA promotes root swelling, and the enlarged roots allow for easier water and nutrient movement as well as root branching [26,27]. In summary, although salt negatively impacts root systems, the roots can adapt to salt stress through muti-gene regulations and hormonal adjustments.

### 2.2. Perception and Response of Salt Stress by Stems

The stem is the main supporting structure of plants, which helps plants grow upright and facilitates the movement of nutrients and water [28]. Plant stems play a crucial function in the uptake of Na^+^ and subsequent transportation to the shoots, enhancing their salt tolerance through a series of adaptive changes [6]. The basic structure of the stem includes epidermal tissue (mainly responsible for protection), vascular bundle tissue (composed of xylem and phloem, with the function of substance transport), fundamental tissue (primarily accountable for storing water and nutrients, while also providing structural support), cambium (located between xylem and phloem, responsible for producing xylem cells inward and phloem cells outward, leading to the gradual thickening of the stem) and pith (with the function of storing nutrients) [29]. 

When exposed to high salinity, plants primarily respond by growing more vascular bundles to stabilize the stem structure and meristem activity, improve salt excretion capacity and ultimately increase salt tolerance (Figure 2). In maize, *ZmLAC9* plays a crucial role in this process, which is mainly expressed in the phloem of vascular bundles as the target gene of microRNA408 (miR408). Under high salt conditions, the overexpression of *ZmLAC9* in plants increases the number of vascular bundle cells. Moreover, miR408 affects the salt tolerance of maize by negatively regulating the expression of *ZmLAC9* [30]. In addition, salt stress can influence the shoot apical meristem (SAM), which is involved in the morphogenesis of plants [31]. Shoot meristemless (STM), a member of the *KNOX1* family, is necessary for SAM development [32]. The formation of STM nuclear condensates is essential for maintaining shoot meristem function in *Arabidopsis* [33]. The prion-like domain (PrD) on STM interacts with the mediator complex subunit 8 (MED8) and BEL1-like (BELL) proteins to form intranuclear aggregates, thereby enhancing its transcriptional activity. Salt treatment can promote the formation of STM aggregates, thereby improving its transcriptional regulation of target genes involved in salt stress response and tolerance and aiding plants in adapting to a saline environment [34].

The mechanical strength of the stem is important in resisting salt stress, which is affected by the accumulation of cell wall lignin [35,36]. The synthesis of lignin is controlled by multiple genes. For instance, MYB46, a transcription factor, can bind to the promoter region of genes related to lignin synthesis, such as *ABRE1A* and *DREB2A*, stimulate the expression of these genes and consequently boost lignin biosynthesis in secondary cell walls of xylem and phloem under salt stress. In transgenic *Arabidopsis* and apple, overexpressing *MdMYB46* exhibits the enhanced tolerance to salt stress [37]. *LAC* genes, which encode laccases, are also involved in the production of lignin. In *Salicornia europaea*, a euhalophyte, overexpression of *SeLAC1* and *SeLAC2* results in an increase in lignin content, secondary cell wall thickness and xylem vessel quantity in transgenic plants [38]. To sum up, the stems of plants mitigate salt stress by stabilizing their own structural integrity, and the maintenance of stem meristem activity plays a crucial role in the process of plant tolerance to salt stress.

### 2.3. Perception and Response of Salt Stress by Leaves

The leaf is the primary site for plant photosynthesis [39]. The fundamental structure of its composition includes the epidermis, which is a protective tissue covering the surface of the leaf and protects internal leaf tissue as well as facilitating gas exchange through stomata; the mesophyll cells, which serve as the primary site for photosynthesis and are normally separated into palisade and spongy tissues; and the veins, which are vascular bundles in leaves that transport water, minerals and organics [40]. Salt stress primarily leads to changes of leaf morphology, chlorosis and stomatal closure, which ultimately affect plant growth and development.

Some genes have been reported to improve the salt tolerance of plants by affecting the morphology and color of leaves (Figure 2). The *Arabidopsis HARDY (HRD)* gene is a AP2/ERF-like transcription factor that enhances the salt tolerance of plants by influencing the structure and function of leaves. When overexpressing *HRD* genes, *Arabidopsis* plants produce smaller and thicker leaves, which is mostly due to more cells forming palisade tissue and sponge tissue [41]. The extended palisade tissue has abundant chloroplasts, and the leaves exhibit a deeper green, which contributes to improving the efficiency of photosynthesis. In barley, knocking out *NUD* (nudum) and *WIN1* (wax inducer 1) genes, which encode the AP2/ERF-type transcription factor of the WIN1/SHN1 subfamily as well, improves the resistance of seedlings to salt stress, and the *win1* knockout lines are manifested as changes in leaf shape and color [42]. Additionally, a rice mutant, *ltr1* (leaf tip rumpled 1), shows wrinkled leaves and is more sensitive to salt stress, demonstrating the role of the rice *LTR* gene in salt resistance [43].

Plants reduce water evaporation by regulating the opening and closing of stomata to adapt to high salt environments. The *SaVHAc1* gene, a vacuolar H^+^-ATPase subunit c1 gene from *Spartina alterniflora*, a halophyte grass, regulates stomatal closure and affects the stomatal density, thus making plants more salt-tolerant [44]. Furthermore, *SaVHAc1* may regulate stomatal opening and closing by affecting the ABA signaling pathway. ABA has been shown to be involved in stomatal closure under salt stress [45]. *OsNAP*, a member of the NAC transcription factor family, can enhance salt tolerance in rice and reduce water loss under salt stress by stimulating ABA-mediated stomatal closure [46]. The constitutive expression of *Gossypium hirsutum WRKY41 (GhWRKY41)* in tobacco enhances stomatal closure in an ABA-dependent manner, thereby improving salt tolerance [47]. In brief, the response of plant leaves to salt is regulated by functional genes which control leaf morphology, color and the opening and closing of stomata.

### 2.4. Perception and Response of Salt Stress by Flowers

As a part of plant reproductive organs, flowers are closely related to the plant salt tolerance. The ability of plants to maintain flower quality and yield under adverse conditions can often be used to assess salt tolerance [48]. The basic structure of a flower includes the sepals (the outermost structure of the flower), the corolla (composed of petals, usually with bright colors and unique shapes), the stamens (the male reproductive organ of the flower, composed of filaments and anthers) and the carpel (forming the pistil and assuming the reproductive function). The flowering time is a key factor for the successful reproduction of the plant. Salinity has a detrimental effect on flowering time, which delays flowering and results in a reduced seed yield [49].

In flowering plants, the processes of pollen germination and pollen tube growth are required for successful fertilization. CPK11 and CPK24 together regulate the activity of the shaker pollen K^+^ channel (SPIK), which is required for pollen germination [50,51]. Furthermore, the timing of flowering and the determination of flower sex are critical elements that contribute to the successful reproduction of plant species. Protein SALT OVERLY SENSITIVE 3 (SOS3) controls flowering through the CONSTANS (CO)-FLOWERING LOCUS T (FT) pathway under salt stress. The GIGANTEA (GI) protein is the main regulator of photoperiod-induced flowering through the CO-FT module. SOS3 promotes nuclear GI protein stabilization under salt stress and prevents delayed flowering. S-acylated SOS3 entering the nucleus interacts with GI and the FLAVIN-BINDING, KELCH REPEAT, F-BOX 1 (FKF1) to regulate CO expression, thereby regulating flowering time under salt stress [49]. E2, an ortholog of *Arabidopsis* thaliana GIGANTEA (GI), is identified as related to the flowering time and salt tolerance of soybean, which delays soybean flowering by enhancing the transcription of the core flowering suppressor gene *E1* and subsequently repressing *FT* expression. An E2 knockout mutant e2^CR^ hydrolyzes H_2_O_2_ and O_2_^−^ into H_2_O and O_2_ to scavenge ROS by releasing peroxidase and therefore enhances salt tolerance in soybean [50]. Additionally, the flowering time and sex of the flower can also be influenced by plant hormones to contribute to salt tolerance (Figure 2). For instance, 135 differentially expressed genes are involved in hormone pathways in the female and male flowers of Jojoba, including the auxin pathway, gibberellin (GA) pathway, abscisic acid (ABA) pathway, cytokinin (CK) pathway and salicylic acid (SA) pathway as well as the jasmonic acid (JA) pathway [51]. Overall, there are many factors involved in the process of flower regulating plant salt tolerance. For successful reproduction in plants, the process of fertilization must be effectively completed. This process is influenced by a range of factors, including pollen germination, the growth of pollen tubes, the timing of flowering and the sex of the flowers, which are impacted by salt stress. Consequently, it is essential to clarify the specific regulatory mechanisms governing these processes under salt stress conditions to enhance plant production.

## 3. Plant Physiological Response to Salt Stress

### 3.1. Salt-Induced Osmotic Stress

**Figure 3:**
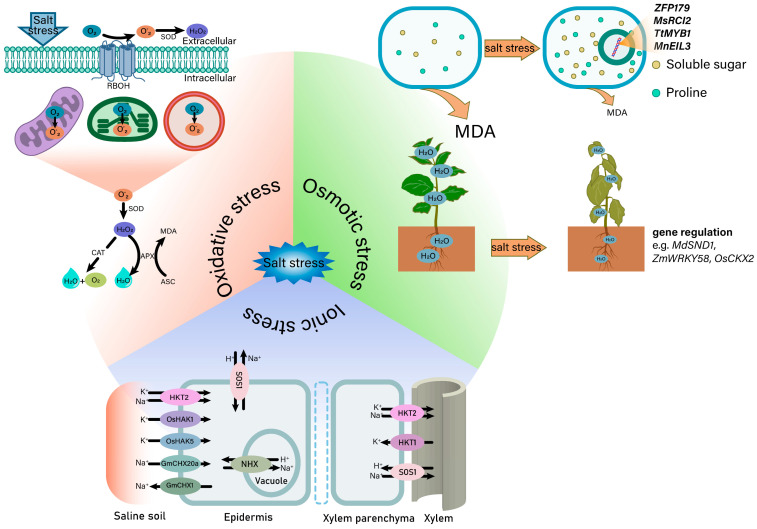
A schematic diagram of plants suffering from osmotic stress, ionic stress and oxidative stress under salt stress. When plants experience osmotic stress due to salt, the soluble sugar content in their cells increases, while RWC and MDA levels decrease. In response to ionic stress caused by salt stress, the concentration of Na^+^ within their cells increases, resulting in a higher Na^+^/K^+^ ratio in the cytoplasm. At this stage, ion transporters play a crucial role in maintaining the Na^+^-K^+^ balance within the cells. Additionally, when plants encounter oxidative stress caused by salt, the levels of ROS in plant cells increase, resulting in membrane lipid peroxidation. Plant cells mitigate ROS through the action of antioxidant enzymes. RWC, relative water content; MDA, malonaldehyde; ROS, reactive oxygen species.

Excessive salt causes plant cells to suffer from osmotic stress, which prevents the roots from absorbing water and ultimately results in a reduction in the relative water content (RWC) of plants [52,53] (Figure 3). In order to cope with water absorption disorders, plants increase the content of osmotic substances in the roots so that the water potential is reduced in the root, thus avoiding root water loss [54]. Soluble sugar and proline are two important osmotic protective substances in plant cells [55]. Furthermore, osmotic stress significantly increased the lipid peroxidation level of the cell membrane and produced malonaldehyde (MDA) [56], which is due to the accumulation of ROS caused by osmotic stress [57]. Therefore, the content of MDA can reflect the ability of plants to resist osmotic stress and further reflects their salt tolerance.

The most direct change caused by osmotic stress is the decrease of the RWC, and the decrease rate is in relation to plant salt tolerance [58,59]. In apple, the leaf RWC of the overexpressing *MdSND1* (a key NAC transcription factor SND1) transgenic plants is higher than that in the wild type after exposure to salt stress [60]. Overexpression of maize *ZmWRKY58* in transgenic rice increases the RWC and enhances the salt tolerance [61]. Additionally, the RWC is also related to the cytokinin content. In rice, the *OsCKX2* knockout mutant exhibits a greater RWC due to its reduced cytokinin levels when exposed to salinity stress [62].

The content of soluble sugar, proline and MDA are affected by muti-genes under salt stress. Overexpression of *ZFP179* (a salt responsive zinc finger protein gene from rice) increases soluble sugar and proline contents and enhances salt tolerance in rice [63]. Similarly, the soluble sugar and proline content of *MsRCI2* (a member of rare cold-inducible 2/plasma membrane protein 3 genes) transgenic alfalfa plants increase significantly compared to the wild type. Overexpression of *MsRCI2A*, *MsRCI2B* and *MsRCI2C* can also improve the salt tolerance of alfalfa [64]. The increase of the osmotic substance content is accompanied by the decrease of the MDA content. For example, overexpression of *TtMYB1* is proved to improve wheat salt tolerance, which contains a higher content of proline and soluble sugar than the wild type in common wheat, while the content of MDA is lower [65]. The overexpression of *MnEIL3* (a gene encoding ethyleneinsensitive3-like proteins) reduces the content of MDA and increases the content of proline under salt stress and finally enhances mulberry salt tolerance [66]. To sum up, in order to cope with osmotic stress caused by salinity, plants usually increase their salt tolerance by adjusting their RWC, the concentration of osmotic adjustment substances and the MDA levels through muti-gene regulations.

### 3.2. Salt-Induced Ionic Stress

Excessive soluble salt in the soil can lead to salt stress, which can cause damage to plants. One of the consequences is ion stress due to an imbalance of ions (Figure 3). During ion stress, Na^+^ accumulates excessively in plants, which interferes with the normal physiological and metabolic processes. Additionally, Na^+^ bears a molecular resemblance to K^+^ and interferes with the uptake of K^+^ by plants, even though Na^+^ cannot fully substitute for the role of K^+^ [67]. Na^+^ can depolarize the membrane potential (MP) depolarization and affect the electrochemical gradient of K^+^, which means that the power of pushing K^+^ into the cell and the K^+^ absorption efficiency are weakened [68,69,70]. However, an intracellular environment with high K^+^ and low Na^+^ levels is more favorable for various biochemical reactions. Simultaneously, maintaining a balance of Na^+^/K^+^ homeostasis within cells is a crucial factor in assessing plant salt tolerance [71]. 

The high-affinity K^+^ (HAK) transporters of rice, *OsHAK1* and *OsHAK5*, mediate the uptake of K^+^ by root epidermal cells and are up-regulated under salt stress [72,73]. Furthermore, high-affinity K^+^ transporters (HKTs) can also mediate the transport of K^+^. This ion-transporter-mediated K^+^ transport is inhibited by Na^+^. However, unlike the former, the process of the HKT uptake of K^+^ is not only inhibited by Na^+^, but also mediates the transport of Na^+^ when the concentration of external Na^+^ is too high [74]. Class 1 HKT proteins (HKT1s) primarily function as Na^+^ selective transporters, while class 2 HKT proteins (HKT2s) primarily serve as Na^+^ and potassium K^+^ transporters [75,76]. Moreover, the CBL-CIPK complex can also mediate the efflux of Na^+^ by activating the SALT OVERLY SENSITIVE 1 (SOS1) protein localized at plasmalemma (PM) under salt stress [77]. Isolation of Na^+^ in the vacuole is also an important way to respond to salt stress, which contributes to maintaining a lower concentration of Na^+^ in the cytoplasm and preventing Na^+^ from harming the enzyme system in the cytoplasm [78]. This process is mainly accomplished by the Na^+^/H^+^ antiporter (NHX) and may be regulated by the SOS pathway [79]. Overexpression of *AtNHX1* in *Arabidopsis* enhances salt tolerance, which is related to NHX1-mediated isolation of Na^+^ in vacuoles [80]. The cation/H^+^-exchanger (CHX) plays multiple roles in plants, including as part of the protective mechanism against salt stress. In soybean, *GmCHX20a* lead to salt sensitivity by increasing Na^+^ uptake by roots, while *GmCHX1* has been shown to protect plants by excluding Na^+^ under salt stress. Therefore, *GmCHX20a* and *GmCHX1* may alleviate osmotic stress and ion stress caused by high salt conditions through a synergistic effect [81]. Taken together, ion transporters play a crucial role in enhancing plant tolerance to salt stress by maintaining a low Na^+^ and high K^+^ environment within the cell.

### 3.3. Salt-Induced Oxidative Stress

Salt stress mainly causes secondary damage to plants, and one of the reasons is the excessive accumulation of ROS induced by salt stress (Figure 3). Because the antioxidant defense in plant cells belongs to the basic metabolism under normal circumstances, the ROS level in the cells is relatively stable. However, salt stress breaks the stable ROS level in cells and causes oxidative damage to plant cells [82]. ROS is mainly produced in chloroplasts, mitochondria and peroxisomes [83]. Common ROS include singlet oxygen (^1^O_2_), hydrogen peroxide (H_2_O_2_), superoxide anions (O_2_^−^) and hydroxyl radicals (OH^•^) [84]. There are many sources of ROS, and the direct cause of O_2_^−^ production is the leakage of electrons to O_2_, especially under salt stress [85]. The production of H_2_O_2_ depends on the membrane-bound NADPH oxidase and apoplastic diamine oxidase activated by salt stress [86,87]. OH^•^ is produced by the interaction of O_2_^−^ with H_2_O_2_ in the Haber–Weiss reaction [88]. The generation of ^1^O_2_ originates from the interaction between photosensitizer molecules activated by light at a specific wavelength and molecular oxygen [89]. 

The scavenging of ROS mainly depends on antioxidant enzymes. It is speculated that some genes control ROS content by changing the related-enzyme activity. For instance, loss of *OsCPK12* affects the activity of the antioxidant enzymes including superoxide dismutase (SOD), peroxidase (POD), catalase (CAT), ascorbate peroxidase (APX) and dehydroascorbate reductase (DHAR), which also causes the accumulation of ROS [90]. *CsBPC2* (*Cucumis sativus* L. BASIC PENTACYSTEINE 2) controls the level of ROS under salt stress by regulating the activity of antioxidant enzymes. In *csbpc2* mutants, SOD, POD, CAT, APX and DHAR have significantly lower activity than that in wild-type plants under saline conditions, with the opposite tendency for H_2_O_2_ and O_2_^−^ levels [91]. Overexpressing *SlER24*, an ethylene-responsive transcriptional co-activator 24 of *Solanum lycopersicum*, reduces ROS accumulation in tomato under salt stress, which has significantly lower levels of H_2_O_2_ and O_2_^−^. Moreover, *SlER24* overexpression in plants is associated with increased SOD and POD activities, suggesting that *SlER24* decreases ROS generation by boosting the activity of antioxidant enzymes [53]. On the other hand, a mutant of *Arabidopsis* lacking cytosolic and/or chloroplastic APX isoforms shows greater salt tolerance in comparison to the wild type [84]. Taken together, the resulting differences in the detoxification of ROS may be due to the uncertain source of ROS under salt stress.

Furthermore, lipid peroxidation caused by salt stress is often used as a marker of salt-induced oxidative membrane damage, which is directly induced by excessive production of ROS [92]. Some substances exert their powerful antioxidant properties by relieving the lipid peroxidation in the membrane lipids. For instance, triacontanol (TRIA), a plant growth regulator, improves cell membrane integrity and lessens salt stress by inhibiting lipid peroxidation in peanut and spinach leaves [93]. MDA is the result of lipid peroxidation and can be used as a standard to measure the degree of lipid peroxidation [94]. When exposed to salt stress, plants overexpressing *FvMYB114*, a *Fragaria vesca* 1R-MYB transcription factor gene, show lower levels of MDA than the wild type and have a reduced degree of membrane lipid peroxidation in transgenic *Arabidopsis*, resulting in greater survival under salt stress [95]. In conclusion, relieving secondary damage caused by salt stress can be achieved through the action of certain antioxidant enzymes.

## 4. Multiple Signaling Pathways Involved in Response to Salt Stress

### 4.1. Mechanisms of Calcium Influx and Signaling Pathway

**Figure 4:**
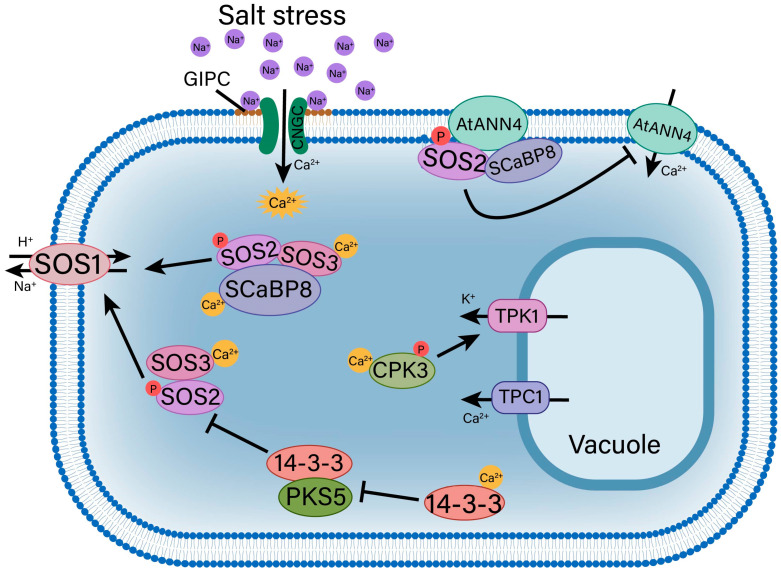
A schematic representation of the Ca^2+^ signaling pathway induced by salt stress. The Na^+^ receptor, GIPC, located on the plasma membrane, activates the Ca^2+^ channel CNGC. This leads to the generation of Ca^2+^ signals within the cell and subsequently triggers a series of signaling pathways. Ca^2+^ in the cytoplasm are recognized by SOS3/SCaBP8, which recruits SOS2 to the plasma membrane to phosphorylate SOS1, facilitating the efflux of Na^+^. Additionally, the CBL-CIPK module plays a crucial role in the SOS pathway. SOS3 interacts with SOS2 upon sensing Ca^2+^, forming the SOS2/SOS3 complex to phosphorylate SOS1 and promote Na^+^ efflux. The activity of SOS2 is negatively regulated by 14-3-3 proteins; however, this inhibitory effect is alleviated by Ca^2+^-mediated binding of PKS5 to the 14-3-3 proteins. TPC1, located on the vacuolar membrane, helps maintain the appropriate balance of Na^+^/Ca^2+^, thereby promoting the proton gradient. Ca^2+^ binding to CPK3 can phosphorylate TPK1 on the vacuolar membrane, ensuring the stable Na^+^/K^+^ in the cytoplasm. Collectively, these ion transporters contribute to the plant tolerance to salt stress. GIPC, glycosyl inositol phosphoryl ceramides; CNGC, cyclic nucleotide gated Ca^2+^ channel; SOS, salt overly sensitive; SCaBP8, SOS3/SOS3-like calcium-binding protein 8; CBL, calcineurin B-like protein; CIPK, CBL-interacting protein kinase; PKS5, SOS2-like protein kinase 5; TPC1, two-pore channel 1; CPK3, calcium-dependent protein kinase 3; TPK1, two-pore K^+^ channel 1.

Calcium (Ca^2+^), as a second messenger, plays a key role in plant response to salt stress [96] (Figure 4). Under saline environments, the binding of Na^+^ to glycosyl inositol phosphoryl ceramides (GIPCs) leads to depolarization of the cell surface potential, which opens Ca^2+^ influx channels and increases Ca^2+^ signal transduction [97]. The two-pore channel 1 (TPC1) protein is very important in long-distance Ca^2+^ signal transduction [98]. Overexpression of *TPC1* enhances nicotinic acid adenine dinucleotide phosphate (NAADP)-mediated calcium signaling [99]. In addition, it is speculated that the highly glycosylated arabinogalactan proteins (AGPs) play a crucial role in Ca^2+^ signaling processes. These proteins not only interact with receptor-like protein kinases but also engage with a variety of other proteins at the junction of the cell wall and plasma membrane, where critical stress signaling events take place [100]. Moreover, AGPs may function as storage molecules that release stored calcium ions in response to salt stress, thereby activating the Ca^2+^-dependent signaling cascade [101].

After entering the cytoplasm, Ca^2+^ signaling is initiated within the cells (Figure 4). The primary objective is to maintain the ratio of cytoplasmic Na^+^ to K^+^, which is essential for achieving salt tolerance in plants. The CBL (calcineurin B-like protein)-CIPK (CBL-interacting protein kinases) signaling system is a crucial part of the SOS pathway, which reacts to Ca^2+^ signals to maintain Na^+^ homeostasis. For instance, SOS3/CBL4 interacts with SOS2/CIPK24 by binding Ca^2+^, and the SOS3/SOS2 complex directly phosphorylates the downstream Na^+^/H^+^ antiporter SOS1 to achieve Na^+^ efflux [102,103]. Similar to this, the CBL8-SOS2/CIPK24-SOS1 module is activated under severe salt stress [104]. Moreover, SOS2-LIKE PROTEIN KINASE5 (PKS5) promotes the interaction between 14-3-3 proteins and SOS2 to inhibit the activity of SOS2. However, Ca^2+^ binds to 14-3-3 proteins to inhibit the kinase activity of PKS5 under salt stress, thereby relieving the inhibitory effect of SOS2 [105]. Salt-induced Ca^2+^ signals are also decoded by SOS3/SCaBP8 (SOS3/SOS3-like calcium-binding protein 8) proteins, which recruit SOS2 to the plasma membrane to phosphorylate and activate SOS1 by interacting with SOS2, thereby increasing Na^+^ efflux [106]. Furthermore, SOS2 phosphorylates AtANNEXIN4 (AtANN4), a family member of Ca^2+^-dependent membrane-binding proteins in plants, reduces its calcium-binding capability, and the interaction between SCaBP8 and AtANN4 inhibits AtANN4-mediated calcium transients, which affects Ca^2+^ perception. This negative feedback loop reduces the level of cytoplasmic sodium in plants and causes a specific and persistent salt stress response [107,108]. CPK3, a family member of CALCIUM-DEPENDENT PROTEIN KINASES (CDPKs) involved in Ca^2+^ signal transduction, is activated by binding to Ca^2+^, thereby phosphorylating and activating the 14-3-3 protein binding site in two-pore K^+^ channel 1 (TPK1). Activated TPK1 binds to growth-regulating factor 6 (GRF6), a 14-3-3 protein, and activates K^+^ channels to maintain the cytoplasmic K^+^/Na^+^ ratio [109]. The importance of the Ca^2+^ signal cannot be ignored, particularly in maintaining Na^+^ homeostasis for salt tolerance. Therefore, it is of great significance to study the mechanism of the Ca^2+^ signal under salt stress for improving the salt tolerance of plants.

### 4.2. Plant Salt Tolerance Mechanism Regulated by Hormonal Signaling

**Figure 5:**
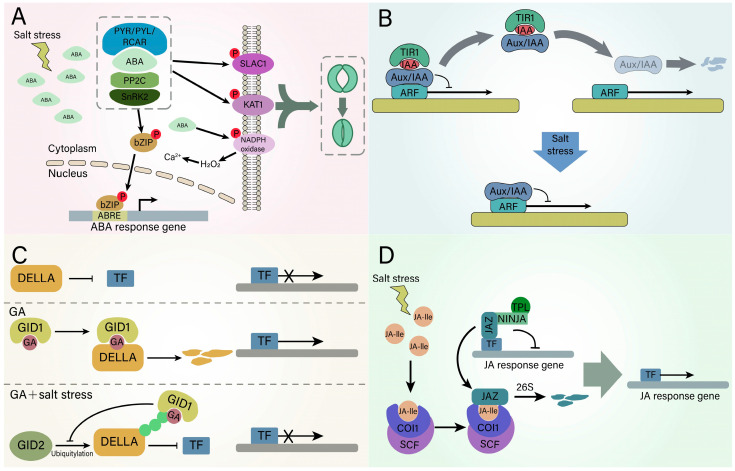
A model of the plant salt tolerance mechanism regulated by hormones under salt stress. (**A**) Salt stress induces an increase in ABA levels. The enhanced binding of ABA to PYR/PYL/RCAR alters its conformation and promotes its interaction with PP2C, thereby inhibiting the activity of PP2C and stimulating the activity of SnRK2. The formation of the PYR/PYL/RCAR-PP2C-SnRK2 complex is a crucial component of the ABA signaling pathway. This complex phosphorylates bZIPs, enhancing its activity. Subsequently, bZIPs translocate to the nucleus to regulate the expression of ABA-responsive genes. Additionally, the complex can phosphorylate SLAC1 and KAT1 on the plasma membrane, facilitating stomatal closure. ABA can also induce stomatal closure through NADPH oxidase-mediated hydrogen peroxide production. (**B**) Under normal conditions, the interaction between IAA and TIR1 leads to the ubiquitination and degradation of Aux/IAA, thereby alleviating the inhibition of Aux/IAA on the activity of the ARF transcription factor. However, under salt stress, the reduced expression of TIR1 prevents the degradation of Aux/IAA, resulting in the continued inhibition of ARF transcription factor activity. (**C**) DELLA acts as a negative regulator in the GA signal transduction pathway. GA forms a complex with GID1 and DELLA, leading to the degradation of DELLA and thereby alleviating its inhibitory effect on GA-responsive genes. Under salt stress, the GA/GID1 complex binds to the N-terminus of DELLA, inducing a conformational change in DELLA that prevents GID2 from binding to it. As a result, DELLA is not degraded, which allows it to maintain its inhibitory effect on gene transcription. The cross means that this process is inhibited. (**D**) JA, induced by salt stress, is recognized by COI1, forming the COI1-JA complex. This complex releases JAZ from its association with co-repressors (NINJA, TPL), which normally inhibit the transcription of JA-responsive genes. Subsequently, JAZ undergoes ubiquitination and is degraded by the 26S proteasome, thereby removing the inhibition on the transcription of JA-responsive genes. ABA, abscisic acid; PP2C, protein phosphatase 2C; SnRK2, sucrose nonfermenting1-related protein kinase 2; SLAC1, slow anion channel 1; KAT1, potassium channel 1; TIR1, transport inhibitor response 1; Aux/IAA, auxin/indole 3-acetic acid; GA, gibberellin; GID, gibberellin insensitive dwarf; JA, jasmonic acid; COI1, coronatine-insensitive 1; JAZ, jasmonate ZIM domain.

The response to salt stress requires the coordination of multiple hormones [110]. Among them, ABA is the most involved in the salt stress response and is the key signal molecule involved in stomatal closure under stress conditions [111] (Figure 5A). In the absence of ABA, PP2C (protein phosphatase 2C) inactivates SnRK2 (sucrose nonfermenting-related protein kinase 2) through direct dephosphorylation [112]. However, to withstand salt stress, plant cells activate the ABA synthesis pathway, leading to elevated ABA levels and the onset of ABA signal transduction [113]. Increased ABA binds to PYR (pyrabactin resistance)/PYL (PYR1-like)/RCAR (regulatory component of ABA receptor), causing an interaction with PP2C, thus releasing the activity of SnRK2 protein kinase. PYR/PYL/RCAR-PP2C-SnRK2, a core complex known as ABA signalosome, can not only regulate the expression of ABA-responsive genes in the nucleus but also phosphorylate ion channels such as SLAC1 (slow anion channel 1) and KAT1 (POTASSIUM CHANNEL 1) on the plasma membrane to induce stomatal closure [114,115,116]. ABA also interacts with components in other signaling pathways. For example, ABA mainly stimulates the production of H_2_O_2_ in guard cells through NADPH oxidase, and the produced H_2_O_2_ rapidly induces an increase of intracellular Ca^2+^ to mediate stomatal closure [117]. However, inhibition of NADPH oxidase-mediated H_2_O_2_ production prevents rapid stomatal closure, leading to a salt-sensitive phenotype [118].

Other plant hormones are also involved in the salt stress response. Auxin binds to TIR1/AFB (TRANSPORT INHIBITOR RESPONSE 1/AUXIN SIGNALING F-BOX), a family member of F-box protein, forming the auxin-TIR1/AFB complex (Figure 5B). This complex promotes the ubiquitination and degradation of auxin/indole 3-acetic acid inducible (Aux/IAA) proteins, thereby activating the downstream auxin response factor (ARF) transcription factors [119]. However, salt stress decreases the expression of the auxin receptor-encoding genes TIR1 and AFB2 [120]. In the absence of gibberellic acid (GA), the DELLA protein interacts with transcription factors and suppresses their activity. When GA is present, the GA receptor gibberellin insensitive dwarf 1 (GID1) combines with the DELLA protein to form a GID1-GA-DELLA complex, which enables the DELLA protein to be ubiquitinated and subsequently degraded [121]. However, GA signaling under salt stress differs from the two cases mentioned above. When GA binds to GID1, a complex known as GA-GID1 attaches to the N-terminus of DELLA. This interaction inhibits the binding of DELLA with GID2 and prevents the ubiquitination process of the DELLA protein. As a result, DELLA dissociates from transcription factor (TF), inhibiting plant growth and development [122] (Figure 5C). At low levels of JA, JAZ proteins are assembled into complexes with co-repressors (such as NINJA, TPL) and TFs to inhibit the expression of JA responsive genes. Under salt stress, plants accumulate bioactive JA molecules, such as JA-lle, and are perceived by the receptor COI1 to form the COI1-JA complex. The JAZ is released from the co-repressor by the complex and is degraded by the 26S proteasome, which relieves the inhibition of JA response genes [123] (Figure 5D). Additionally, ethylene can induce ROS detoxification mechanisms and promote plant survival under salt stress [124]. Salicylic acid (SA) is involved in the accumulation of osmoprotectants, induction of antioxidant enzymes and improvement of ion homeostasis under salt stress [125,126,127]. In summary, plants exhibit a complex hormonal response to salt stress, and the intricate interactions among these hormones allow plants to adapt effectively to saline environments.

Plants build a defense system by orchestrating the synthesis, signaling and metabolism of various hormones via multiple crosstalks [128]. Firstly, the synthesis and metabolic pathways of different hormones can influence each other. The production of cytokinins (CKs) in roots and their transportation through the xylem are detrimental to plants experiencing salt stress. Under such conditions, an increase in ABA levels correlates with a decrease in CK levels in tomatoes [129]. Reduced CK levels heighten the sensitivity of plants to ABA, leading to a decrease in branch growth. This adaptive response enables plants to survive in stressful environments [130]. In cotton, salt stress strongly induces the expression of the zinc finger transcription factor *GhPLATZ1*. Ectopic expression of *GhPLATZ1* in *Arabidopsis* resulted in faster germination of *Arabidopsis* than wild type by inhibiting the transcription of *ABI4* [131]. *ABI4* has been reported to regulate the transcription of GA catabolic gene *GA2ox7* and ABA synthesis gene *NCED6* [132]. Secondly, different hormone-induced signaling pathways also have interactions. For example, the application of ABA can significantly increase the number of LRs in ABA receptor mutants *pyl8* and *pyl9* [133]. Further studies showed that PYL8/9 promoted the initiation of LR primordia (LRP) by regulating the MYB77-ARF7-mediated signaling module [134]. The application of exogenous auxin supplemented the phenotype of a low LR number in *pyl8/9*, suggesting that ABA-induced LRP initiation may be auxin-dependent [133]. Similarly, salt-induced strigolactone (SL) production mediated the up-regulation of ABA biosynthetic gene *LsNCED2* in mycorrhizal lettuce roots, thereby increasing the ABA content [135]. On the contrary, ABA also affected the SL concentration and signal transduction in arbuscular mycorrhizal (AM) Sesbania cannabina seedlings by up-regulating *CCD7*, *CCD8* and *MAX2* [136].

### 4.3. Phosphatidic Acid (PA) Signaling in the Regulation of Salt Stress

**Figure 6:**
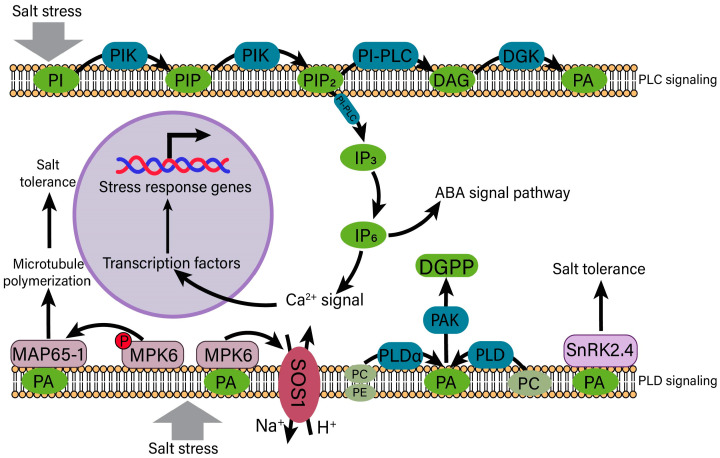
A model of PA signaling in plant cells induced by salt stress. Salt stress is detected at the cell membrane, activating the PLC and PLD signaling pathways. In the PLC pathway, PI is sequentially phosphorylated to form PIP and PIP_2_. Upon cleavage by PI-PLC, PIP_2_ is converted into DAG and IP_3_. DAG can then be transformed into PA in the presence of DGK, while IP_3_ diffuses into the cytoplasm and is further converted into IP_6_. IP_6_ plays a crucial role in the ABA signaling pathway, influencing the release of Ca^2+^ and subsequently modulating the expression of salt stress-responsive genes. In the PLD pathway, PA can be generated from the hydrolysis of PC by PLD or from the hydrolysis of both PC and PE by PLDα. Through the action of PAK, the resulting PA can induce the formation of DGPP, a signaling molecule in plant cells. Alternatively, MPK6 can bind to PA to activate SOS1 on the plasma membrane, facilitating the expulsion of Na^+^. MPK6 can also phosphorylate MAP65-1 bound to PA, enhancing microtubule polymerization in response to salt stress. Additionally, SnRK2.4 can promote salt tolerance in plants by binding to PA. PA, phosphatidic acid; PLC, phospholipase C; PLD, phospholipase D; PI, phosphoinositides; PIP, phosphatidylinositol 4-phosphate; PIP_2_, phosphatidylinositol 4,5-bisphosphate; DAG, diacylglycerol; IP_3,_ inositol 1,4,5-triphosphate; DGK, diacylglycerol kinase; IP_6,_ hexakisphosphate; PC, phosphatidylcholine; PE, phosphatidylcholine; PAK, PA kinase; DGPP, diacylglycerol pyrophosphate; MPK6, mitogen protein kinase 6; MAP65-1, microtubule-associated protein 65-1.

Phospholipase C (PLC) and phospholipase D (PLD) signaling pathways are triggered when the cell membrane senses salt stress [137] (Figure 6). Phosphoinositide-specific phospholipase C (PI-PLC) catalyzes the hydrolysis of phosphatidylinositol-4,5-bisphosphate (PIP_2_), which is formed when phosphoinositides (PI) is sequentially phosphorylated into phosphatidylinositol 4-phosphate (PIP) and PIP_2_, to generate soluble second messenger inositol 1,4,5-triphosphate (IP_3_) and diacylglycerol (DAG) [138]. IP_3_ diffusion into the cytoplasm is converted to inositol hexakisphosphate (IP_6_), and they directly or indirectly trigger the release of Ca^2+^, thereby influencing the gene response to salt stress through transcription factors [139]. In addition, IP_6_ plays a role in ABA regulation of Ca^2+^ release from guard cells [140]. DAG is converted to phosphatidic acid (PA) in the action of diacylglycerol kinase (DGK) [141]. The aforementioned describes one of the pathways responsible for the production of PA, namely, the PLC pathway.

Moreover, PLD can also hydrolyze structural phospholipids, such as phosphatidylcholine (PC) and phosphatidylethanolamine (PE), into PA [142] (Figure 6). PA can also be produced via hydrolysis of phospholipids on the terminal phosphodiester bond by PLD and further phosphorylate PA via PA kinase (PAK) to generate diacylglycerol pyrophosphate (DGPP), a signaling molecule in plant cells [141]. One of the downstream targets of PA is mitogen protein kinase 6 (MPK6), which harbors two main functions. Firstly, MPK6 phosphorylates microtubule-binding protein (MAP65-1) and increases microtubule polymerization under salt stress conditions [143]. The binding of MAP65-1 to PA with PLD1 increases MAP65-1 activity and thereby promotes microtubule stability under salt stress conditions. In addition, PA and MAP65-1 interact with MPK6 in plant cells, which is induced by NaCl. The *mpk6* mutant exhibits reduced microtubule depolymerization in pavement cells and is sensitive to salt stress [144]. Secondly, the kinase activity of MPK6 is activated to phosphorylate SOS1 to regulate Na^+^ homeostasis [96]. Two SnRK2 protein kinases (SnRK2.4 and SnRK2.10), identified as PA-binding proteins, are recruited to the cell membrane under salt stress [145]. However, PA does not directly influence the activity of SnRK2 kinases, while PABD/domain 1 in SnRK2.4 is shown to play a role in the salt stress response in *Arabidopsis* [146,147]. Briefly, PA, as a signal molecule, activates the downstream salt stress response and crosstalk with other signaling pathways primarily via two ways through salt induction.

### 4.4. ROS Signaling and Homeostasis under Salt Stress

**Figure 7:**
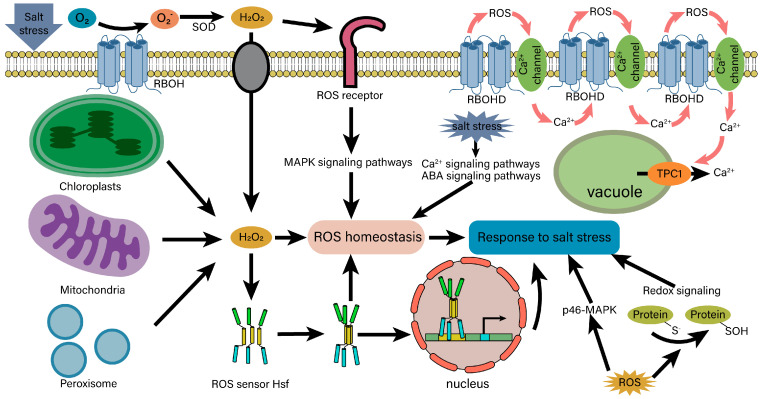
A schematic diagram of ROS signal transduction induced by salt stress. When plant cells are exposed to salt stress, they generate a significant amount of ROS, which can lead to oxidative damage. To mitigate this damage, plant cells maintain ROS homeostasis through various signal transduction pathways activated by salt stress. Under normal conditions, ROS, such as H_2_O_2_, are typically produced by chloroplasts, mitochondria and peroxisomes. Additionally, H_2_O_2_ can be generated from extracellular oxygen through the action of RBOH and SOD under salt stress conditions. The produced H_2_O_2_ is subsequently transported into the cell or detected by ROS receptors on the cell membrane, which activates the MAPK signaling pathway to help maintain intracellular ROS homeostasis. Excessive H_2_O_2_ in the cytoplasm is recognized by ROS sensors, triggering downstream signal transduction pathways. For instance, H_2_O_2_ interacts with the ROS sensor Hsf to form a homotrimer that translocates to the nucleus, where it activates the expression of genes associated with oxidative stress. Furthermore, ROS can also be detected by other ROS sensors, such as p46-MAPK, to facilitate the plant response to salt stress. Additionally, ROS signaling can interact with other signaling pathways, highlighting the complexity of stress responses in plants. For example, ROS generated by RBOHD stimulate the opening of Ca^2+^ channels in the plasma membrane, leading to an influx of calcium ions, which generates Ca^2+^ signals. Subsequently, this influx activates TPC1 on the vacuolar membrane, allowing Ca^2+^ from the vacuole to enter the cytoplasm, which further activates RBOHD. This cycle can create a ROS/Ca²⁺ wave, thereby enhancing the salt tolerance of plants. RBOH, respiratory burst oxidase homolog; SOD, superoxide dismutase; MAPK, mitogen-activated protein kinase; Hsf, heat shock factor; RBOHD, respiratory burst oxidase homolog D.

The mitogen-activated protein kinase (MAPK) cascade is an important component in the ROS signaling pathway [148] (Figure 7). In *Arabidopsis*, the MEKK1-MKK1/MKK2-MPK4 cascade is a key regulator of ROS stress signal transduction, which controls the activity of ROS scavenging enzymes to maintain ROS homeostasis and responds to salt stress [149]. MPK3/6 is another crucial element of ROS signal transduction. In the salt intolerance 1 (SIT1)-MPK3/6 cascade, SIT1 activates MPK3/6 and promotes the accumulation of ethylene and ROS, thereby regulating rice salt response [150]. OXI1, a serine/threonine kinase necessary for signal transduction mediated by an oxidative burst, increases the salt tolerance of *Arabidopsis* through the oxidative signal-inducible1 (OXI1)-MPK3/6 cascade [151]. Notably, the ROS signaling pathway can crosstalk with multiple other signaling pathways. Under salt stress, the ROS produced by RESIRATORY BURST OXIDASE HOMOLOG D (RBOHD) diffuses through the apoplast and activates ROS-sensitive Ca^2+^ channels on the plasma membrane, thereby triggering a Ca^2+^ influx and mediating the release of additional Ca^2+^ activated by TPC1 from the vacuole. This process further enhances the Ca^2+^ signal and activates more RBOHD proteins, ultimately resulting in the formation of ROS/Ca^2+^ waves, which promotes the systemic response of *Arabidopsis* roots to salt stress [152]. Additionally, ROS is produced by *AtRBOHD* and *AtRBOHF* in the NADPH pathway and limits the speed of ABA signal transduction [153]. Salt-induced ABA and Ca^2+^ signaling pathways can fine-tune the activity of AtRBOHF by activating the SnRK2.6 and CIPK11/26 signaling pathway modules, thereby modulating ROS homeostasis in plant responses to high salinity [154,155].

There are several ROS sensors/receptors in plant cells, with heat shock factors (HSFs) acting as the notable ones. Two putative models for *HSFs* functioning in plants are proposed [156] (Figure 7). (i) In *Arabidopsis*, AtHsfA4a or AtHsfA2a, form a homotrimer after interaction with ROS and are transported to the nucleus to activate the expressions of oxidative stress-related genes. (ii) Oxidative stress induces the homotrimerization of a specific HSF and interacts with another HSF, mediating its transport to the nucleus. These two active HSFs then cooperate to induce gene expression. Alternatively, ROS-mediated activation of a HSF is able to activate ROS scavenging and detoxification enzymes, such as APX and SOD, to reestablish the homeostasis of cellular ROS levels [157]. In addition, a 46 kDa phospho-p38-like MAPK (p46-MAPK) functions as a putative sensor of the redox status, which activates and initiates downstream signaling events such as microtubule disruption and assembly of non-canonical tubulin polymers [158]. The cysteine residues can act as sensors for redox changes, triggering different post-translational modifications and in turn modulating redox-dependent pathways to respond to salt stress [159]. Some ROS-scavenging enzymes also function as redox receptors, detecting the intracellular redox state. For example, peroxiredoxins (PRDXs) play a crucial role in redox signaling by serving as redox sensors and regulating the binding affinity of chaperonins [160]. 

Overall, the elevated levels of ROS under salt stress serve as signals that are detected by ROS sensors and receptors, which then transduce these signals to regulate plant responses to salt stress. These redox sensors play a crucial role in detecting disturbances in ROS homeostasis. Additionally, the MAPK signaling pathway is a key component of ROS signal transduction, contributing to the regulation of ROS homeostasis. Together, these elements form a signaling network that enables plants to effectively respond to salt stress.

## 5. Molecular Breeding of Plants with Salt Tolerance

### 5.1. The Role of Genomics in Salt-Tolerant Breeding

**Figure 8:**
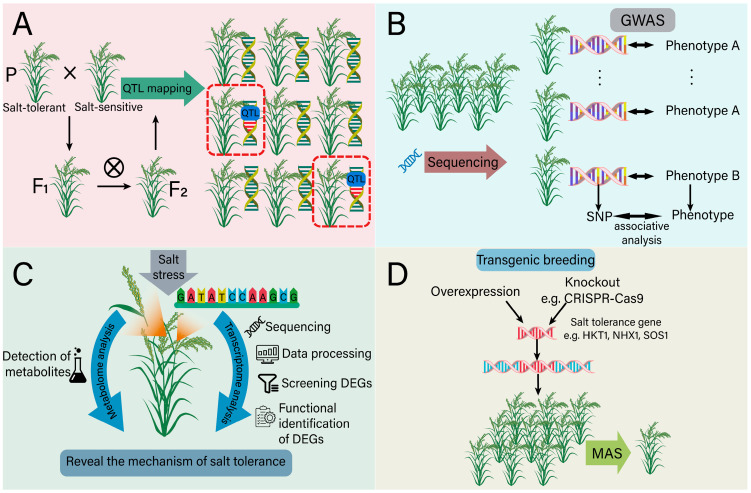
A schematic diagram of the strategies using molecular technologies for breeding salt-tolerant crops. Salt-tolerant genes are identified through (**A**) QTL mapping population, (**B**) GWAS population and (**C**) metabolomics and transcriptomics analysis. (**D**) Plants with salt tolerance can be acquired via transgenic breeding, including gene overexpression and knockout techniques. Furthermore, the superior alleles of genes can be developed into a functional molecular marker, aiding in the breeding of salt-tolerant plants through MAS. QTL, quantitative trait locus; GWAS, genome-wide association study; MAS, marker assisted selection. The red dotted box represents the selected salt-tolerant plants. A cross with a black circle indicates self-intersection. The cross without a black circle indicates hybridization.

A molecular marker, based on the nucleotide sequence variation between individuals, has more stable and observable advantages in comparison with traditional markers [161]. With the development of molecular marker technology, marker-assisted selection (MAS) technology has been widely used in crop breeding, which provides a new way to accelerate the genetic improvement of crops with salt tolerance [162]. The quantitative trait loci (QTL) related to salt tolerance are of great significance for salt-tolerance breeding, and their identification relies on marker-assisted breeding (MAB) (Figure 8A). MAB helps to capture natural variations in chromosome regions and uses DNA markers linked to the specific QTL to select genotypes with the required alleles [163]. In cucumber, Liu et al. used a recombinant inbred line (RIL) population and identified the locus of qST6.2 related with salt tolerance [164]. In the genomics region of this QTL, *Csa6G487740* and *Csa6G489940* were further identified to be associated with cucumber salt tolerance, which may contribute to the development of salt-tolerant cucumber varieties through MAS [164]. Similarly, the QTL conferring soybean salt tolerance were detected using mapping populations crossed by the varieties Cheongja 3 (salt sensitive) and IT162669 (salt tolerant), and two novel major loci (qST6 and qST10) were identified using a high-density genetic map [165]. Eight putative candidate genes, including phosphoenolpyruvate carboxylase and an ethylene response factor, were finally screened in association with ion toxicity and physiological responses to salinity [165]. Some other QTL related with plant salt tolerance have also been identified, such as qSIS2, qWCSST2, qST12.3, qGR6.2, qST1.2 and qST6 in rice, as well as qSNAX.2A.1 and qSNAX.2A.2 in wheat (Table 1). In a recent study, 12 major QTL associated with salt tolerance in *Brassica napus* L. were successfully identified using high-throughput quantitative trait locus sequencing (QTL-seq). Combining the QTL-seq data with transcriptome analysis on the parent lines under salt stress, 10 salt-tolerant candidate genes were selected, and notably, a single nucleotide polymorphism (SNP) marker located in the *BnaC04g40820D* was found to be associated with salt tolerance and can be used as a diagnostic marker for MAB [166].

The combination of QTL and a genome-wide association study (GWAS) can effectively reduce the probability of false positive markers and contributes to identifying multiple genes that are associated with salt tolerance [175]. On the basis of high-throughput sequencing and SNP markers, GWAS has become an effective tool for analyzing the genetic structure of salt tolerance traits in various plant species [176] (Figure 8B). By scanning the whole genome, GWAS can detect the associations between genetic variations and specific traits. Breeders can select desirable traits more accurately and minimize the co-transfer of the linked undesirable traits. For example, Kumar et al. used 220 rice samples to identify the salt tolerance gene loci and detected a total of 20 SNPs significantly associated with leaf Na^+^/K^+^ and 44 SNPs associated with other salt tolerance parameters [177]. Based on 6,361,920 SNPs of 478 rice accessions, Shi et al. obtained 22 salt tolerance-related SNPs according to salt tolerance-related traits [178]. Lekklar et al. identified 146 genes co-localized with previously reported salt QTL in 104 rice accessions using GWAS [179]. Under salt stress, a total of 51,060 SNPs distributed among 26 chromosomes were screened using GWAS among cotton accessions at the seedling stage, and finally, 27 SNPs were detected that are significantly associated with three salt tolerance-related traits [180]. Moreover, GWAS has predicted a new minor locus for salt tolerance on soybean chromosome 8 and identified several SNPs that are significantly associated with soybean salt tolerance [181]. In conclusion, the identification of SNPs or QTL through genomics applications offers a foundational theoretical framework for MAS in breeding salt-tolerant plants [182]. 

Epigenetics provides an explanation for the phenotypic differences observed among organisms with identical genomes. It involves various epigenetic factors, such as histone modification and DNA methylation, which regulate gene expression [183]. Notably, epigenetic mechanisms play a crucial role in enhancing plant salt tolerance. For instance, histone acetyltransferase GCN5 (general control non-repressed protein 5) promotes cell wall integrity and salt tolerance by activating certain cellulose synthesis genes, such as *CTL1* (chitinase-like gene), *PGX3* (polygalacturonase involved in expansion 3) and *MYB54* (MYB domain protein 54). This process is associated with GCN5-mediated acetylation of histone H3 at lysine 9 (H3K9) and lysine 14 (H3K14) [184]. Epigenetics plays a crucial role in generating stress memories that enable plants to better withstand future stress exposure [185]. Chemical modifications of histones are key mechanisms in regulating the salt stress response in plants. For instance, primed soybean seedlings exhibit distinct transcriptome profiles, with changes in histone marks such as histone 3 lysine 4 dimethylation (H3K4me2), histone 3 lysine 4 trimethylation (H3K4me3) and histone 3 lysine 9 acetylation (H3K9ac). These histones altered the salt stress response through modification ion homeostasis, cell wall and defense-related transcriptional network [186]. This highlights how plants can adapt to their environment through epigenetic changes, providing valuable insights for breeding salt-tolerant varieties.

### 5.2. The Role of Transcriptome in Salt-Tolerant Breeding

Transcriptome analysis plays a central role in detecting gene expression and transcriptional regulation, providing insight into how plants adapt to salt stress environments [187,188] (Figure 8C). Through comprehensive detection of RNA transcripts in cells or tissues under specific physiological conditions, transcriptome analysis unveils the dynamic changes in gene expression in plants under salt stress [189]. This provides a robust tool for comprehending the mechanisms of salt tolerance and breeding for salt-tolerant crops. In soybean seedlings, transcriptome analysis revealed significant changes in 1235 differentially expressed genes (DEGs) under salt stress conditions, including 116 differentially expressed transcription factors (TFs), of which 17 TFs belong to the MYB family [190]. The expression level of *GmMYB46* was significantly up-regulated under salt stress, and the overexpression experiments confirmed that *GmMYB46* enhances salt tolerance in transgenic *Arabidopsis*, primarily by activating the expression of salt stress response genes encoding delta1-pyrroline-5-carboxylate synthase 1 (P5CS1), SOD and 9-cis-epoxycarotenoid dioxygenase 3 (NCED3) [190]. Transcriptome analysis of peanuts also demonstrates the complexity of gene expression under salt stress. It is worth noting that two genes encoding ω-3 fatty acid desaturase are down-regulated under the treatment of 250 mM sodium chloride for 4 days and up-regulated after 3 days of transferring under standard conditions, indicating lipid metabolism plays an important role in plant adaptation to salt stress [191]. In transcriptome analysis of *BpPP2C1* transgenic plants, a large number of DEGs related to salt stress are enriched in the ABA signaling pathway, the flavonoid biosynthesis pathway, oxidative stress and anion transport, indicating that *BpPP2C1* influences the plant salt stress response by regulating these key biological processes [192]. In rapeseed, a total of 18,040 DEGs have been identified through transcriptome analysis under salt stress [193]. Among them, the down-regulated DEGs are primarily associated with hormone signal transduction pathways, photosynthesis and transcription factors, while the up-regulated genes are linked to amino acid biosynthesis and ion transport. The additional enrichment of DEGs identified under stress through transcriptome analysis is presented, such as the ABA signaling pathway in rice, the MAPK signaling pathway in maize and the carbohydrate, glutamine and xyloglucan metabolic pathways in *Podocarpus Macrophyllus* (Table 2). Moreover, the detection of long non-coding RNAs (lncRNAs) provides insights into how plants respond to salt stress [194]. Specifically, lncRNA TCONS_00116877 is located approximately 3.9 kb upstream of *Medtr7g094600*, which encodes glutathione peroxidase and is up-regulated in roots. This suggests that TCONS_00116877 may play a role in regulating plant tolerance to oxidative stress by modulating the expression of POD [195]. In addition, *Medtr1g081900*, regulated by lncRNA TCONS_00020253, encodes a vacuolar Na^+^/H^+^ antiporter, which facilitates Na^+^ influx into vacuoles and is induced by salt stress [195]. Taken together, these findings demonstrate that the transcriptome can not only reveal the significance of specific genes involved in metabolic and signaling pathways in plant salt tolerance mechanisms, but also offer a solid theoretical foundation for developing salt-tolerant crop varieties through molecular breeding techniques. 

### 5.3. The Role of Metabolome in Salt-Tolerant Breeding

As a potent tool for revealing metabolic states of organisms, metabolomics is becoming more and more significant in the study of plant salt tolerance [206] (Figure 8C). Metabolites are low molecular weight compounds produced by metabolics in living organisms, and the metabolic processes constantly change over the time. During plant evolution, plant-specific metabolites have been greatly diversified, especially under salt stress, and they are considered to be key players in the complex interactions between plants and their environments [207]. Several metabolites have been shown to enhance salt tolerance in plants, providing a strong rationale for utilizing metabolome analysis. For instance, flavonoids are prevalent secondary metabolites found in plants. Metabolomic analysis revealed that differentially accumulated metabolites (DAMs) in alfalfa under NaCl stress were primarily enriched in pathways related to isoflavone biosynthesis, flavonoid biosynthesis, ABC transporters, α- linolenic acid metabolism and anthocyanin biosynthesis [208]. Under salt stress, the expression of the flavonoid hydroxylase (F3H) gene was upregulated, promoting the accumulation of flavanols and enhancing antioxidant enzyme activity in tobacco [209]. Dihydroflavonol reductase (DFR) genes within the anthocyanin biosynthetic pathway regulate anthocyanin accumulation, which contributes to improving salt tolerance in *Brassica napus* [210]. Overexpression of the flavonol synthase (FLS) gene can increase the flavonoid content and consequently enhance salt tolerance in plants [211]. Metabolomics analysis shows that many metabolites produced by plants, exposed to salt stress, are involved in osmotic regulation and osmotic protection [212]. Taking salt-tolerant sesame for example, it is found that its metabolic group show significant changes under salt stress, especially the enhancement of amino acid and sucrose metabolic pathways, which indicates that plants improve their resistance to salt by adjusting the levels of specific metabolites [213]. Similarly, in wild soybeans and gramineous plants, enhancement of the tricarboxylic acid (TCA) cycle has been shown to increase energy reserves and intermediate metabolite levels, thereby enhancing plant salt tolerance [214]. Other metabolic pathways associated with salt stress are also listed, such as secondary metabolism in rice and amino acid metabolism, fatty acid metabolism and sugar alcohol metabolism in *Soja* (Table 2). Additionally, in a recent study of rice, the metabolome data revealed the diversity and complexity of metabolite accumulation in different tissues [215]. Taken together, metabolomics analysis can identify metabolites that contribute to plant tolerance to salt stress, offering potential metabolic engineering goals for breeding more salt-tolerant crop varieties.

### 5.4. Transgenic Breeding

Transgenic breeding, a contemporary biotechnology method, has shown significant potential in producing new lines of salt-tolerant crops in response to the increasingly serious global problem of soil salinity [216]. In order to prove the effect of genes on the regulation of salt tolerance in plants, gene overexpression and knockout are usually employed to accurately verify gene function (Figure 8D). The overexpression of specific functional genes has significantly improved crop salt tolerance. For example, overexpressing genes of *HKT1*, *NHX1* or *SOS1* confer salt tolerance in transgenic *Arabidopsis* and tomato under salt stress [217]. Overexpressing *OsSOS2* shows enhanced salt tolerance via maintaining favorable ion homeostasis [218]. When *ZmWRKY58* is heterogeneously expressed in rice, the transgenic plants exhibit more tolerance to salt stress [219]. Conversely, gene knockout studies highlight the significance of genes involved in salt tolerance in the opposite way. The *AtSPT4-2* (a transcription elongation factor suppressor of Ty 4-2) knockout mutant exhibits a salt sensitive phenotype [220]. *OsNAC041* knockout rice is sensitive to salt, which is associated with the changes in expression of genes related with ROS signaling [221]. Knocking out *OsbHLH024* in rice using the CRISPR/Cas9 system can enhance the expression of the ion-related genes *OsHKT1;3*, *OsHAK7* and *OsSOS1*, thereby improving the salt tolerance of rice [222]. Other studies on plant salt tolerance achieved through transgenic technology have also been presented, such as the knock-out of *OsRR22* in rice, the overexpression of *GmNHX5* in soybean and the overexpression of *AtNAC2* in transgenic *Arabidopsis* plants (Table 3). It seems to be that mining and verifying new salt-tolerant genes through the two transgenic methods will lay the foundation for the cultivation of new salt-tolerant crops in the future.

More importantly, transgenic technology is a key link in translating basic scientific research achievements into practical applications [236]. Through transgenic technology, salt tolerance genes obtained via QTL, GWAS, transcriptome and metabolome analysis can be introduced into the genome of the target crop, thus conferring stronger salt tolerance to the crop. In addition, the combination of molecular marker-assisted breeding and transgenic breeding can accelerate the process of salt tolerance breeding. In transgenic breeding, molecular markers can be used to screen plants that successfully integrate exogenous genes to ensure that the selected plants can stably express the desired traits, which provides valuable resources for MAS for breeding crops with salt tolerance (Figure 8D). 

## 6. Future Prospects

To sum up, it is evident that various plant tissues exhibit distinct responses to salt stress. Nevertheless, this subject has received limited attention in the past. Therefore, we primarily focus on the specific responses of different plant tissues to salinity, providing valuable insights for future research on the mechanisms underlying salt stress responses. Meanwhile, considerable advances have been achieved in comprehending how plants sense and react to salt stress (Na^+^) and transport Na^+^ ions; the molecular mechanisms underlying plant salt tolerance have also been uncovered. The genomic approaches, including GWAS and QTL analysis, as well as multi-omics methods such as transcriptomics and metabolomics, are high-efficiency technologies for identifying genes associated with salt tolerance. This paper also indicates that these genes regulate plant salt tolerance through various mechanisms, including hormone regulation and the balance of Na^+^ and K^+^ levels, highlighting their potential application in molecular breeding of salt-tolerant crops. However, there are still some restrictions regarding current studies, as well as difficulties for future research.

(1) Excavating new genes associated with salt tolerance from the abundant wild resources.

At present, soil salinization poses a great threat to agriculture, so it is urgent to continually excavate more novel salt-tolerant genes. Wild resources have accumulated abundant genetic diversity during long-term natural selection and evolution, especially for the genes related to abiotic stresses. Through screening and identification, these genes can be applied to crop breeding to effectively improve the salt tolerance of crops. However, wild resources primarily grow in diverse environments, and the environmental differences may affect the salt tolerance of plants. Therefore, it is necessary to consider the interaction between tolerant genes and environmental conditions, which makes the regulation mechanism of salt tolerance in plants more complicated.

(2) Exploring the roles of plant hormones in plant salt tolerance.

As important signal molecules in plants, plant hormones play a key role in regulating plant growth, development and response to environmental stress. Under salt stress, a series of complex physiological and biochemical changes occur in plants, which is inseparable from the comprehensive regulation of plant hormones. However, despite the fact that numerous plant hormones have been preliminarily identified involving in the salt response or in signaling regulation, only analysis of ABA signaling has been extensively conducted. Therefore, it becomes essential to deeply explore other hormone signaling pathways related to salt tolerance and the complex interplay among various plant hormones. The core genes involved in the hormonal process that respond to salt stress can be utilized in crop genetic improvement, ultimately providing a theoretical foundation for the breeding of salt-tolerant crops.

(3) Developing novel phenotype methods to better observe how plants maintain a balance between salt tolerance and growth.

Monitoring how plants balance salt tolerance and growth is helpful to understand the survival mechanism of plants under salt stress and evaluate their salt tolerance so as to formulate effective strategies to improve the crop yield under salt stress. Traditional methods face many challenges in assessing crop salt tolerance, such as the long cycle and low efficiency. The establishment of new phenotype approaches, such as multi-spectral data from unmanned aerial vehicles, can quickly and accurately evaluate the salt tolerance of plants to guide the breeding of salt-tolerant crops. Due to the integration of plant growth and development status, physiological and biochemical indicators, as well as gene expression and other multifaceted information in these phenotype techniques, greatly enhance the accuracy and reliability of crop salt tolerance assessment compared to relying solely on a single morphological index. This approach provides a comprehensive evaluation of plant salt tolerance and growth, giving insightful guidance for developing effective strategies to enhance crop yield.

(4) Application of multi-omics technology.

With the rapid advancement of next-generation sequencing (NGS), multi-omics technology has emerged as an effective approach for crop improvement, enabling the identification and interpretation of stress responses in various crops. The integration of multi-omics techniques can address some limitations associated with the single-omics method, providing a more comprehensive understanding of gene functions and regulatory networks under environmental stress conditions. For instance, combining metabolomics and transcriptomics analysis can establish a metabolite–gene association network and identify candidate genes involved in metabolic pathways. Moreover, delving into the emerging omics technologies, such as single-cell and spatial transcriptomes, and integrating genomic, transcriptomic, proteomic, metabolomic and phenomic data from a broader range of crop lines will enable a systematic identification of genes and superior variations that enhance crop salt tolerance. Indeed, integrating data from various platforms and formats and analyzing and interpreting the resulting information poses a considerable challenge. However, it is a necessary step for gaining a comprehensive understanding of the factors that impact crop salt tolerance.

(5) Using molecular markers to breed salt-tolerant crop varieties.

Salt tolerance in plants is a complex trait regulated by multiple QTL/genes. To efficiently and accurately identify individuals with salt tolerance genes, molecular marker technology is primarily utilized to screen a specific genotype of the offspring populations. Recently, numerous high-throughput molecular markers have evolved as a powerful tool for breeding programs and variety development, which has significantly reduced the breeding cycle and accelerated the entire breeding process.

## Figures and Tables

**Table 1:** Some QTL associated with plant salt tolerance.

Species	Parents	Traits	QTL	Reference
Rice (*Oryza Longistaminata*)	9311 × wild rice	Salt injury score and water content of seedling	qSIS2, qWCSST2	[167]
Rice (*Oryza sativa*)	Huazhan × Nekken2	Germination ability under salt stress	qST12.3	[168]
Rice (*Oryza sativa*)	Wujiaozhan × Nipponbare	Percentage of germination under salt stress	qGR6.2	[169]
Rice (*Oryza sativa*)	Dongxiang/Ningjing 15 × Ningjing16	Salt tolerance of rice at seedling stage	qST1.2, qST6	[170]
Wheat (*Triticum aestivum* L.)	WTSD91 × WN-64	Na^+^ exclusion ability	qSNAX.2A.1, qSNAX.2A.2	[171]
Wheat (*Triticum aestivum* L.)	Excalibur×Kukri	Maintenance of shoot growth under salinity, Na^+^ accumulation, Cl^−^ accumulation, K^+^/Na^+^ ratio	QG_(1-5)_.asl-5A, QG_(1-5)_.asl-7B, QNa.asl-2A, QCl.asl-2A, QCl.asl-3A, QK:Na.asl-2DS2	[172]
Zoysiagrass (*Zoysia Japonica*)	Z105 × Z061	Salt tolerance traits	qLF-1, qLF-2, qSCW-1	[173]
Cotton (*Gossypium hirsutum* L.)	GX1135 × GX100-2	Yield component traits under salt stress	qLY-Chr6-2, qBNP-Chr4-1, qBNP-Chr12-1, qBNP-Chr15-5, qLP-Chr19-2, qLP-Chr5-3, qLP-Chr13-1, qBW-Chr5-5	[174]

**Table 2:** Biological processes associated with salt stress through transcriptome and metabolome analyses.

Species	Omics Method	Biological Processes Associated with Salt Stress	Reference
Rice (*Oryza sativa*)	Transcriptome	ROS homeostasis, ABA signaling pathway and osmotic and ionic homeostasis	[196]
Rice (*Oryza sativa*)	Transcriptome	ABA signaling pathway	[197]
Maize (*Zea mays* L.)	Transcriptome	The MAPK signaling pathway—plant and plant hormone signal transduction	[198]
Sesame (*Sesamum indicum* L.)	Transcriptome	Oxidation-reduction process and oxidoreductase activity	[199]
*Podocarpus Macrophyllus*	Transcriptome	The carbohydrate, glutamine, and xyloglucan metabolic pathways	[200]
Rice (*Oryza sativa*)	Metabolome	Secondary metabolites such as aminoadipic acid, calactin and satratoxin H and glycerylphosphorylethanolamine	[201]
Canola (*Brassica napus*)	Metabolome	Lipid metabolism	[202]
Soja (*Glycine soja*)	Metabolome	Amino acid metabolism, fatty acid metabolism, sugar alcohol metabolism, carboxylic acids, the TCA cycle, antioxidants from secondary metabolism and nucleic acids	[203]
Halophytic Grass (*Puccinellia nuttalliana*)	Metabolome	Proline, dopamine, phosphatidylcholines and the enriched TCA cycle in leaves	[204]
Foxtail millet (*Setaria italica* L.)	Metabolome	The biosynthetic pathways of phenylpropanoids, flavonoids, lignin and lysophospholipids	[205]

**Table 3:** Application of transgenic technologies in salt-tolerant genes.

Gene Name	Molecular Strategy	Functions	Reference
*GmNHX5*	Overexpression	Maintaining higher K^+^/Na^+^ ratio	[223]
*PcCFR*	Overexpression	Keeping the photosynthetic cycle by unabated generation of RuBP and retaining better light harvesting capacity of the leaves under stress	[224]
*TdPIP2*	Overexpression	Reducing water evaporation from leaves	[225]
*TaNIP*	Overexpression	Regulating the balance of Na^+^ and K^+^	[226]
*MdSUT2.2*	Overexpression	Scavenging of ROS and transporting sucrose	[227]
*IbPSS1*	Overexpression	Maintaining Na^+^ homeostasis	[228]
*OsRR22*	Knock-out	Increasing plant height and total fresh weight	[229,230]
*ZmHKT1*	Knock-out	Promoting Na^+^ exclusion of leaves	[231]
*BEARI*	Knock-out	Regulating the expression of salt-responsive genes and ions transport	[232]
*OsNAC45*	Overexpression and knock-out	Regulating germination and seedling growth	[233]
*OsmiR535*	Overexpression and knock-out	Improving resistance to NaCl	[234]
*AtNAC2*	Overexpression and knock-out	Promoting the development of lateral roots	[235]

## Data Availability

Not applicable.

## Abbreviations

ABA, abscisic acid; AFB3, auxin signaling F-Box 3 receptor; AGPs, highly glycosylated arabinogalactan proteins; AM, arbuscular mycorrhizal; APX, ascorbate peroxidase; ARF, auxin response factor; Aux/IAA, auxin/indole 3-acetic acid; BELL, BEL1-like; bHLH, basic helix-loop-helix; Ca^2+^, calcium; CAT, catalase; CBL, calcineurin B-like protein; CDPKs, CALCIUM-DEPENDENT PROTEIN KINASES; CHX, cation/H^+^-exchanger; CIPK, CBL-interacting protein kinases; CK, cytokinin; CO, CONSTANS; CsBPC2, Cucumis sativus L BASIC PENTACYSTEINE 2; CYP79B2, cytochrome P450 family 79 subfamily B2; DAG, diacylglycerol; DAMs, differentially accumulated metabolites; DEGs, differentially expressed genes; DFR, dihydroflavonol reductase; DGK, diacylglycerol kinase; DGPP, diacylglycerol pyrophosphate; DHAR, dehydroascorbate reductase; EC, electrical conductivity; F3H, flavonoid hydroxylase; FKF1, FLAVIN-BINDING, KELCH REPEAT, F-BOX 1; FLS, flavonol synthase; FT, FLOWERING LOCUS T; GA, gibberellin; GCN5, general control non-repressed protein 5; GI, GIGANTEA; GID1, gibberellin insensitive dwarf 1; GIPCs, glycosyl inositol phosphoryl ceramides; GRF6, growth-regulating factor 6; GWAS, genome-wide association study; HAK, high-affinity K^+^ transporter; HKT, high-affinity K^+^ transporter; HRD, HARDY; HSFs, heat shock factors; IP_3_, inositol 1,4,5-triphosphate; IP_6_, inositol hexakisphosphate; JA, jasmonic acid; KAT1, POTASSIUM CHANNEL 1; lncRNAs, long non-coding RNAs; LR, lateral root; LRP, LR primordia; ltr1, leaf tip rumpled 1; MAB, marker-assisted breeding; MAP65-1, microtubule-associated protein 65-1; MAPK, Mitogen-activated protein kinase; MAS, marker-assisted selection; MDA, malonaldehyde; MED8, mediator complex subunit; miR408, microRNA408; MP, membrane potential; MPK6, mitogen protein kinase 6; NCED3, 9-cis-epoxycarotenoid dioxygenase 3; NGS, next-generation sequencing; NHX, Na^+^/H^+^ antiporter; OXI1, oxidative signal-inducible1; p46-MAPK, 46 kDa phospho-p38-like MAPK; P5CS1, delta1-pyrroline-5-carboxylate synthase 1; PA, phosphatidic acid; PAK, PA kinase; PC, phosphatidylcholine; PE, phosphatidylethanolamine; PI, phosphoinositides; PIN2, PIN-FORMED2; PIP, phosphatidylinositol 4-phosphate; PIP_2_, phosphatidylinositol-4,5-bisphosphate; PI-PLC, phosphoinositidespecific phospholipase C; PKS5, SOS2-LIKE PROTEIN KINASE5; PLC, phospholipase C; PLD, phospholipase D; PM, plasmalemma; POD, peroxidase; PP2C, protein phosphatase 2C; PR, primary root; PrD, prion-like domain; PRDXs, peroxiredoxins; PYL, PYR1-like; PYR, pyrabactin resistance; QTL, quantitative trait locus; QTL-seq, quantitative trait locus sequencing; RBOHD, respiratory burst oxidase homolog D; RCAR, regulatory component of ABA receptor; RIL, recombinant inbred line; ROS, reactive oxygen species; RWC, relative water content; SA, salicylic acid; SAM, shoot apical meristem; SCaBP8, SOS3-like calcium-binding protein 8; SIT1, salt intolerance 1; SL, strigolactone; SLAC1, slow anion channel 1; SNPs, single nucleotide polymorphisms; SnRK2, sucrose nonfermenting-related protein kinase 2; SOD, superoxide dismutase; SOS1, SALT OVERLY SENSITIVE 1; SOS3, SALT OVERLY SENSITIVE 3; SPIK, shaker pollen K^+^ channel; STM, shoot meristemless; TCA, tricarboxylic acid; TF, transcription factor; TIR1/AFB, TRANSPORT INHIBITOR RESPONSE 1/AUXIN SIGNALING F-BOX; TPC1, two-pore channel 1; TPK1, two-pore K^+^ Channel 1.
